# Promoting AMPK/SR-A1-mediated clearance of HMGB1 attenuates chemotherapy-induced peripheral neuropathy

**DOI:** 10.1186/s12964-023-01100-9

**Published:** 2023-05-04

**Authors:** Xing Yang, Rumeng Jia, Fan Hu, Wen Fan, Tongtong Lin, Xiaotao Zhang, Chenjie Xu, Shirong Ruan, Chunyi Jiang, Yan Li, Cailong Pan, Yang Yang, Liang Hu, Qi Chen, Wen-Tao Liu

**Affiliations:** 1 Department of Pharmacology, School of Basic Medical Sciences, Nanjing Medical University, Nanjing, 211166 China; 2 Department of Radiation Oncology, Qingdao Central Hospital, Qingdao, 266042 Shandong China; 3 Department of Anesthesiology and Pain, Nanjing First Hospital, Nanjing Medical University, Nanjing, 210006 Jiangsu China; 4 Department of Oncology, Shandong Provincial Qianfoshan Hospital, The First Hospital Affiliated with Shandong First Medical University, Jinan, 250014 Shandong China; 5 Department of Anesthesiology, The Affiliated Cancer Hospital of Nanjing Medical University & Jiangsu Cancer Hospital & Jiangsu Institute of Cancer Research, Nanjing, 210009 China; 6 Atherosclerosis Research Center, Key Laboratory of Cardiovascular Disease and Molecular Intervention, Nanjing Medical University, Nanjing, 211166 Jiangsu China

**Keywords:** Macrophage, Clearance, SR-A1, HMGB1, CIPN

## Abstract

**Background:**

Chemotherapy-induced peripheral neuropathy (CIPN) is a serious side effect of chemotherapy with poorly understood mechanisms and few treatments. High-mobility group box 1 (HMGB1)-induced neuroinflammation is the main cause of CIPN. Here, we aimed to illustrate the role of the macrophage scavenger receptor A1 (SR-A1) in HMGB1 clearance and CIPN resolution.

**Methods:**

Oxaliplatin (L-OHP) was used to establish a CIPN model. Recombinant HMGB1 (rHMGB1) (his tag) was used to evaluate the phagocytosis of HMGB1 by macrophages.

**Results:**

In the clinic, HMGB1 expression and MMP-9 activity were increased in the plasma of patients with CIPN. Plasma HMGB1 expression was positively correlated with the cumulative dose of L-OHP and the visual analog scale. In vitro, engulfment and degradation of rHMGB1 increased and inflammatory factor expression decreased after AMP-activated protein kinase (AMPK) activation. Neutralizing antibodies, inhibitors, or knockout of SR-A1 abolished the effects of AMPK activation on rHMGB1 engulfment. In vivo, AMPK activation increased SR-A1 expression in the dorsal root ganglion, decreased plasma HMGB1 expression and MMP-9 activity, and attenuated CIPN, which was abolished by AMPK inhibition or SR-A1 knockout in the CIPN mice model.

**Conclusion:**

Activation of the AMPK/SR-A1 axis alleviated CIPN by increasing macrophage-mediated HMGB1 engulfment and degradation. Therefore, promoting HMGB1 clearance may be a potential treatment strategy for CIPN.

**Video abstract**

**Supplementary Information:**

The online version contains supplementary material available at 10.1186/s12964-023-01100-9.

## Background

Chemotherapy-induced peripheral neuropathy (CIPN) is a major dose-limiting side effect of chemotherapeutic agents, including platinum drugs, taxanes, epothilones, and vinca alkaloids [1]. Up to 76.5% of patients develop CIPN during and after treatment with chemotherapeutic drugs [2], especially oxaliplatin (up to 89%) [3]. The manifestation is peripheral neuropathy with a ‘stocking and glove’ distribution, characterized by sensory loss, paresthesia, dysesthesia, numbness, and tingling, often aggravated by neuropathic pain [4]. Unfortunately, there is no effective treatment to date [5]. Thus, there is an urgent need to explore the underlying mechanisms and develop effective treatment strategies.

Neuroinflammation is widely considered the major mechanism of CIPN [6, 7]. Excessive expression of pro-inflammatory cytokines (TNF-α, IL-6, IL-1β, etc.) causes peripheral sensitization and subsequent central sensitization, leading to CIPN [8, 9]. High mobility group box 1 (HMGB1), a damage-associated molecular pattern (DAMP), can induce the expression of pro-inflammatory cytokines and act as an initiator and amplifier of neuroinflammation in neuropathic pain [10, 11]. Our previous study demonstrated that oxaliplatin induced the release of HMGB1 from neurons and Raw 264.7 cells, which activates MMP-9 and induces and aggravates CIPN [12], suggesting that HMGB1 could be a potential target for CIPN therapy.

Endocytosis of ligand molecules is considered a mechanism of signal attenuation via receptor and ligand clearance from the cell surface [13]. Macrophage scavenger receptor A1 (SR-A1), a member of the scavenger receptor family, plays multiple roles in the phagocytosis of macrophages [14, 15]. A previous study showed that SR-A1 helped clear DAMPs in the brain to prevent excess inflammation after ischemic stroke [16]. The scavenger receptor SCARA5 could act as an HMGB1 recognition molecule that is negatively involved in HMGB1-mediated inflammation in fish models [17]. However, the relationship between SR-A1 and HMGB1 in CIPN models has not yet been reported.

AMP-activated protein kinase (AMPK) is an evolutionarily conserved sensor of cellular energy status [18]. Our previous study showed that AMPK activation significantly alleviated chronic constriction injury-induced neuropathic pain [19]. Evidence also indicates a novel role of AMPK in modulating the phagocytic activity of macrophages [20]. However, the effect of AMPK on HMGB1 clearance has rarely been reported. Thus, we hypothesized that AMPK activation may promote the engulfment of HMGB1 and alleviate CIPN, in which SR-A1 is involved.

For the first time, we demonstrated that activation of the AMPK/SR-A1 axis promotes engulfment and degradation of HMGB1 to attenuate CIPN. We also provided a potential therapeutic agent, metformin, for CIPN treatment.

## Materials and methods

### Patients and clinical data

To explore the critical role of HMGB1 in the development of CIPN, we collected plasma samples from cancer patients with or without chemotherapy (chemotherapy regimen containing oxaliplatin) provided by Nanjing First Hospital. The research was a self-control study, which was approved by the institutional review board of Nanjing First Hospital, registered under KY20171228-KS-01. All methods were performed in accordance with relevant guidelines and regulations. Twenty patients were enrolled in the study. The baseline characteristics of the study population are summarized in Additional file 1: Table S1. The median (range) age of the patients was 63.5 (34–83) years, and 58.3% of patients were male. The median (range) total oxaliplatin (L-OHP) dose was 1200 mg/m^2^. The inclusion criteria were as follows: (1) Karnofsky score > 50%; (2) expected survival time > 3 months; (3) at least one measurable lesion confirmed by CT or PET-CT; (4) normal bone marrow reserve and heart, liver, and kidney function; (5) no serious complications and no second primary tumors; and (6) oxaliplatin or oxaliplatin combination therapy used as chemotherapeutic agents.

Moreover, pain intensity was measured using a Visual Analog Scale (VAS). VAS is most commonly a straight 100 mm line, without demarcation, with the words “no pain” at the left-most end and “worst pain imaginable” (or something similar) at the rightmost end. Patients were instructed to place a mark on the line indicating the amount of pain they felt at the time of evaluation. The distance of this mark from the left end was then measured, and this number was used as a numerical representation of the severity of the patient's pain. The median (range) of the VAS score was 5 (2–10). HMGB1 concentration in patients’ plasma was measured by ELISA. The correlation between VAS and L-OHP dose, plasma HMGB1 accumulation and L-OHP dose, and VAS and plasma HMGB1 accumulation in CIPN patients were analyzed using linear regression analysis. We found that plasma HMGB1 accumulation is positively correlated with VAS and L-OHP cumulative dose in patients with CIPN.

### Human ethics statement

All procedures were performed in strict accordance with the regulations of The Ethics Committee of the International Association for the Study of Pain.

### Animal ethics statement

All animal experiments were approved by the Nanjing Medical University Animal Care and Use Committee (no. IACUC-1908026) and were designed to minimize suffering and the number of animals used. All procedures were performed in strict accordance with the regulations of the Guide for the Care and Use of Laboratory Animals (The Ministry of Science and Technology of China, 2006).

### Animals

Adult male ICR mice (18–22 g) were provided by the Experimental Animal Center of Nanjing Medical University, Nanjing, China. SR-A1 KO mice (C57BL/6 background) were generated according to a previously described method, and SR-A1 WT mice with identical genetic backgrounds were used as controls. Animals were housed five to six per cage under pathogen-free conditions with soft bedding at a controlled temperature (22 ± 2 °C) and a 12 h light/dark cycle (lights on at 8:00 a.m.). Behavioral testing was performed during the light cycle (between 9:00 a.m. and 5:00 p.m.). Animals were allowed to acclimate to these conditions for at least 2 days before inclusion in the experiments. For each experimental group, the animals were matched for age and body weight.

### Reagents and antibodies

β-actin antibody was purchased from Santa Cruz Biotechnology (Santa Cruz, CA, USA). His-HMGB1 and Transferrin antibodies were purchased from Abcam (Cambridge, MA, USA). Phosphorylated PKC (Thr514) and phosphorylated AMPK (Thr172) antibodies were purchased from Cell Signaling Technology (Beverly, MA, USA). SR-A1 antibody was purchased from R&D Systems (Minneapolis, MN, USA). The secondary antibodies for western blotting were purchased from Sigma-Aldrich (St. Louis, MO, USA). Human HMGB1/HMG1 Protein (his tag) was purchased from Sino Biological (China). AMPK agonists (AICAR, A-769662, Metformin) and antagonists (Compound C), PKC inhibitor (Go 6983), chloroquine, and endocytosis inhibitors (chlorpromazine, genistein, and amiloride) were purchased from MCE (China). TLR4 inhibitor (Cli-095), Lipopolysaccharide (LPS), Dextran sulfate sodium salt, Dimethyl Sulfoxide (DMSO), and MG132 were purchased from Sigma-Aldrich (St. Louis, MO, USA). The RAGE inhibitor (FPS-ZM1) was purchased from Calbiochem. Recombinant Murine IFN-γ was purchased from PeproTech. Fucoidan was purchased from APExBIO (USA). Normal mouse IgG and anti-SRAI mouse polyclonal IgG were purchased from R&D Systems (Minneapolis, MN, USA). Fetal bovine serum (FBS) and other cell culture media and supplements were purchased from Gibco (Grand Island, NY, USA). Immunofluorescent antibodies for c-fos and CGRP were purchased from Cell Signaling Technology (Beverly, MA, USA). The secondary antibodies for immunofluorescence were purchased from Jackson ImmunoResearch Laboratories (West Grove, PA, USA) and Abcam (Cambridge, MA, USA).

### Bone-marrow-derived macrophage (BMDM) isolation and culture

BMDMs were harvested from the femurs and tibia of the naive C57BL6/J mice or SR-A^−/−^ mice (Details were shown in the figure legends of each experiment), and differentiated in Dulbecco’s modified Eagle’s medium (DMEM) supplemented with 10% (v/v) FBS, penicillin (100 U/mL), streptomycin (100 U/mL) (KeyGEN), and 10% L929 cell supernatant for 7 days. On day 7, BMDMs were washed and seeded in 6-well tissue culture plates. The cells were activated with LPS (E. coli 0111:B4;100 ng/ml; Sigma) + IFN-γ (50 ng/ml; Peprotech) for 24 h.

### Isolation of human peripheral blood mononuclear cells (PBMC)-derived macrophages

Primary human monocytes were isolated from the whole blood of cancer patients before L-OHP treatment. PBMCs were isolated by gradient centrifugation in a Ficoll-Paque PREMIUM sterile solution (GE Healthcare). Monocytes were purified using CD14 microbeads according to the manufacturer’s instructions (Invitrogen) and then were treated with 5 ng/ml recombinant human granulocyte–macrophage colony-stimulating factor (GM-CSF, switching monocytes to M1 phenotype macrophages), and cultured at 37 °C and 5% CO_2_ for 5 days in the RPMI 1640 medium with 10% fetal bovine serum (FBS, Gibco).

### Assay for internalization of HMGB1

BMDMs were incubated with recombinant human HMGB1 protein fused with a polyhistidine tag (10 nM; Sino Biological) in the presence or absence of AICAR (300 μM; MCE) and then analyzed by western blotting. To inhibit internalization, the SR-A1 inhibitor dextran sulfate sodium salt (100 μg/ml; Sigma), AMPK inhibitor Compound C (20 μM; MCE), and PKC inhibitor Go6983 (100 nM; MCE) were added 15 min prior to stimulation with AICAR. To track the internalized protein in lysosomes, HMGB1-FITC and LysoTracker™ Red DND-99 (75 nM; Invitrogen) were added to co-culture with BMDMs for 60 min at 37 °C. After incubation for 60 min, the cells were washed with phosphate-buffered saline (PBS), followed by nuclear staining with Hoechst 33,342 (10 μg/ml; Invitrogen) for 5 min. The cells were then observed using confocal microscopy (Carl Zeiss LSM710 confocal system).

### Behavioral analysis

Animals were habituated to the testing environment daily for at least two days before baseline testing. Mechanical sensitivity was detected using Von Frey Hairs (Woodland Hills, Los Angeles, CA, USA). Animals were placed in boxes set on an elevated metal mesh floor and allowed to habituate for 30 min before testing. The plantar surface of each hind paw was stimulated with a series of von Frey hairs with logarithmically increasing stiffness perpendicular to the plantar surface. Each mouse was tested three times, and the average threshold was measured.

### Western blotting

Samples (cells and spinal cord tissue segments at L4‒L6, sciatic nerve, or dorsal root ganglion (DRG)) were collected and washed with ice-cold PBS before being lysed in a lysis buffer. Then, the sample lysates were separated by SDS-PAGE and electrophoretically transferred onto polyvinylidene fluoride membranes (Millipore). The membranes were blocked with 10% whole milk in TBST (Tris·HCl, NaCl, and Tween 20) or 5% bovine serum albumin (BSA) in TBST for 2 h at room temperature and then probed with primary antibodies at 4 °C overnight. The primary antibodies used were p-AMPK (Thr172), 1:1000; HMGB1, 1:1000; p-PKC (Thr514), 1:1000; his tag, 1:1000; β-actin, 1:1000; SR-A1, 1:500; and transferrin, 1:1000. The membranes were developed using enhanced chemiluminescence reagents (Millipore). Subsequently, bands were detected using horseradish peroxidase (HRP)-coupled secondary antibodies. Data were acquired with a Molecular Imager (ChemiDoc, Bio-Rad) and analyzed using ImageJ software (National Institute of Health, USA).

### Gelatin zymography

Animals were deeply anesthetized with sodium pentobarbital (40 mg/kg). Orbital blood samples were collected and centrifuged (3500 rpm, 5 min), and the supernatant was stored at – 80 °C after L-OHP administration (except where otherwise stated) under a microscope and homogenized in 1% NP40 lysis buffer. A total of 300–500 μg of protein per lane was loaded into the wells of precast gels (8% polyacrylamide gel containing 0.1% gelatin). For in vitro experiments, the cell culture medium was collected 4 h after L-OHP treatment. Fifty microliters of medium per lane were loaded into the wells of precast gels (8% polyacrylamide gels containing 0.1% gelatin). After electrophoresis, each gel was incubated with 50 mL of developing buffer for 48 h (37.5 °C) in a shaking bath. The gels were then stained with Coomassie brilliant blue (1%, with 10% acetic acid and 10% isopropyl alcohol, diluted with dd H_2_O).

### Cell staining

BMDMs were plated in glass bottom cell culture dishes and activated with LPS (E. coli 0111:B4; 100 ng/ml; Sigma) + IFN-γ (50 ng/ml; Peprotech) for 24 h. The cells were then treated with or without AICAR (300 μM) for 15 min before being co-cultured with Human HMGB1 protein (his tag) (10 nM). BMDMs were fixed with 4% paraformaldehyde and permeabilized with 0.3% Triton X-100. After blocking with 10% donkey serum in PBS for 2 h, the coverslips with BMDM cells were incubated at 4℃ with the His Tag antibody (1:200) diluted in PBS overnight. The coverslips were then exposed to fluorescent secondary antibody (1:300, at room temperature for 2 h) and rinsed three times with PBS. 4′,6-Diamidino-2-phenylindole (DAPI) is a fluorescent DNA dye that marks the nucleus. Confocal microscopy was performed using a Carl Zeiss LSM710 confocal system.

### Immunofluorescence assay

Under deep anesthesia by an intraperitoneal injection of sodium pentobarbital (40 mg/kg), the animals were perfused transcardially with normal saline followed by 4% paraformaldehyde in 0.1 M PB, pH7.4, each for 20 min. Then, L4 and L5 lumbar segments were dissected and post-fixed in 4% paraformaldehyde. The embedded blocks were sectioned at a thickness of 25 μm. Sections from each group (five animals in each group) were incubated with rabbit antibodies for c-Fos (1:200) and CGRP (1:200). The free-floating sections were washed with PBS and incubated with secondary antibody (1:300; Jackson Laboratories, USA) for 2 h at room temperature. After washing three times with PBS, the samples were examined under a confocal microscope (Olympus FV1000 confocal system, Olympus, Japan) for morphological details of immunofluorescence staining. The examinations were performed blindly. Images were randomly coded, and fluorescence intensities were analyzed using ImageJ software. The average green fluorescence intensity of each pixel was normalized to the background intensity of the same image.

### Quantitative PCR (qPCR)

Total RNA was extracted from BMDMs using TRIzol reagent (Invitrogen, CA, USA). Isolated RNA was reverse transcribed into cDNA using HiScript II Q RT SuperMix for qPCR (Vazyme Biotech Co., Ltd.) following the standard protocol. Quantitative real-time PCR (qPCR) was performed using ChamQ SYBR qPCR Master Mix (Vazyme Biotech Co., Ltd.) with a QuantStudio 5 Real-Time PCR Detection System (Thermo Fisher Scientific). The relative expression levels of TNF-α, IL-1β, and IL-6 were calculated and quantified using the 2 − ΔΔCt method after normalization with a reference. All the primers used are listed in Additional file 1: Table S2.

### Determination of platinum concentration in DRG

The procedure was according to the instruction. In short, DRG samples were collected on the 7 th day after the first administration of L-OHP (3 mg/kg, *i.p.*) or vehicle (n = 4). Nitric acid (analytical purity) was added into the DRG samples to completely dissolve the samples (37 ℃ water bath for 10 min). Hydrogen peroxide was added into the solution (room temperature for 10 min). Inductively coupled plasma emission mass spectrometer (ICP-MS 7800) was started to detect platinum content in samples and collect and analyze data.

### Statistics

GraphPad Prism 6 software (GraphPad Software, San Diego, CA, USA) was used to conduct all the statistical analyses. Alteration of expression of the proteins and the behavioral responses were tested with one-way analysis of variance (ANOVA), and the differences in latency over time among groups were tested with two-way ANOVA, followed by Bonferroni’s post hoc tests. Unpaired Student’s t-test was used to analyze differences between the two groups. The correlation between visual analog scale (VAS) and L-OHP dose, plasma HMGB1 accumulation and L-OHP dose, and VAS and plasma HMGB1 accumulation in CIPN patients were analyzed using linear regression analysis. Results are expressed as mean ± SEM. *p* values < 0.05 were considered significant.

## Results

### L-OHP-induced CIPN is associated with a high level of HMGB1 accumulation and MMP-9/2 activity

**Fig. 1:**
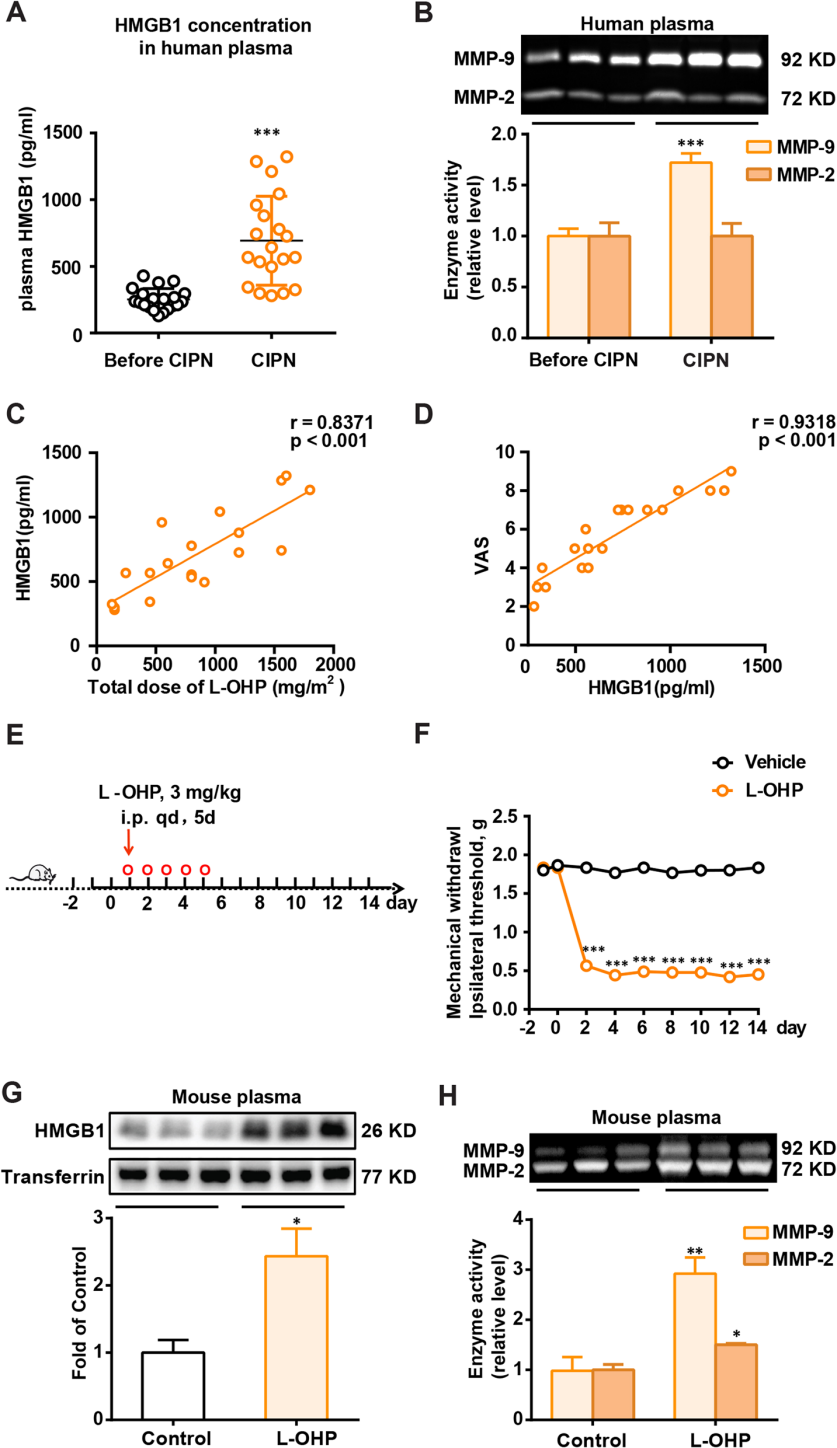
HMGB1 accumulation and MMP-9/2 activity are increased in the plasma of CIPN patients and mice. **A**, **B** Plasma HMGB1 content (n = 20) and MMP-9 (n = 12) activity in cancer patients before L-OHP treatment (Control) and after the last L-OHP treatment (CIPN). The correlation between plasma HMGB1 and L-OHP dose (**C**) and plasma HMGB1 and VAS (**D**), respectively. VAS was performed after the last L-OHP treatment (n = 20). **E** The general scheme of experiments in vivo. **F** Mechanical thresholds of mice received L-OHP administration (3 mg/kg, *i.p.*) or vehicle for five consecutive days (n = 6). **G** and **H** HMGB1 content and MMP-9/2 activities in plasma of mice measured by western blotting and Gelatin zymography (n = 3). Plasma was collected from mice on the 14th day after the first administration of L-OHP (3 mg/kg, *i.p.*) or vehicle. Representative bands and a data summary (n = 3 for each group) are shown. A significant difference was revealed following unpaired Student’s t-test (A, B, G and H) or two-way ANOVA (F) or Linear regression analysis (C and D) (*p < 0.05, **p < 0.01 and ***p < 0.001 vs. Control; Bonferroni post hoc tests)

To explore the critical role of HMGB1 in the development of CIPN, we evaluated the concentration of HMGB1 and the activity of MMP-9 in the plasma of patients with CIPN and mice. MMP-9 is a downstream target enzyme of HMGB1 and is an important marker of pain. As shown in Fig. 1A and B, HMGB1 concentration and MMP-9 activity in the plasma of patients significantly increased after L-OHP treatment. There is a positive correlation between visual analogue scale (VAS) and L-OHP dose (r = 0.8594, *p* < 0.001, data not show), plasma HMGB1 accumulation and L-OHP dose, VAS and plasma HMGB1 accumulation in CIPN patients respectively (Fig. 1C and D). Additionally, mice were treated with L-OHP (3 mg/kg, *i.p.*, five consecutive days at a total dose of 15 mg/kg) to establish CIPN mice models(Fig. 1E). L-OHP significantly decreased the mechanical thresholds of the mice on day 2, and the induced allodynia lasted until day 14 (Fig. 1F). Compared to the vehicle group, HMGB1 accumulation and MMP-9 activity were dramatically increased in CIPN mouse plasma (Fig. 1G and H). These data suggest that excessive accumulation of HMGB1 plays a vital role in CIPN development.

### AMPK activation increases macrophages’ engulfment of HMGB1 and reduces inflammation

**Fig. 2:**
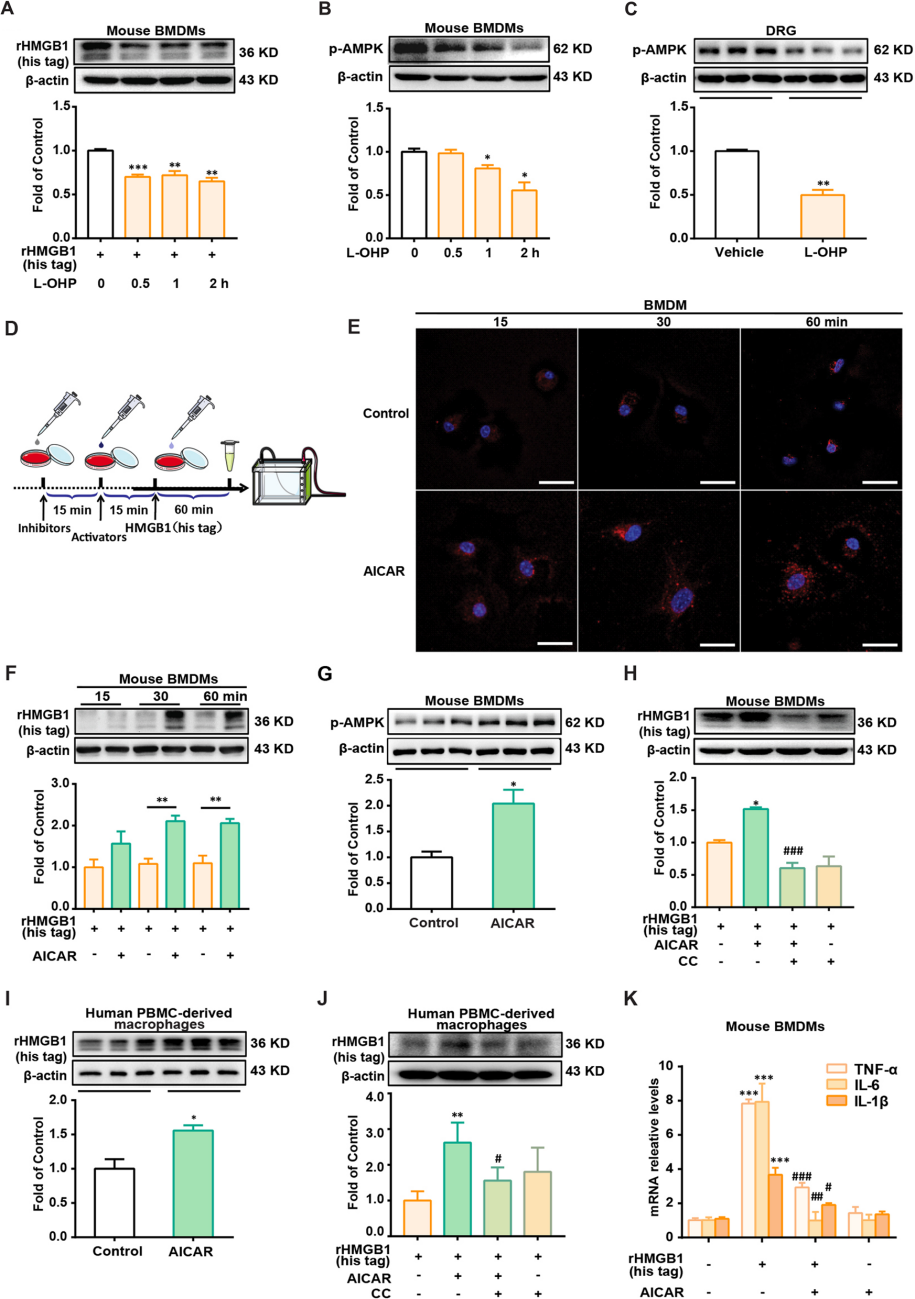
AICAR increases the engulfment of HMGB1 and reduces inflammation in mouse BMDMs and human PBMC-derived macrophages. **A** rHMGB1 (his tag) content in BMDMs treated with L-OHP (1 μM). Cells were co-cultured with rHMGB1 (his tag) (10 nM) for 1 h after L-OHP administration (n = 3). **B** Phosphorylation of AMPK in BMDMs treated with L-OHP (1 μM) (n = 3). **C** Phosphorylation of AMPK in mice DRG. DRG samples were collected on the 14th day after the first administration of L-OHP (3 mg/kg, *i.p.*) or vehicle (n = 3). **D** The general scheme of experiments in vitro. Confocal microscopy images (red) **E** or content **F** of rHMGB1 (his tag) in BMDMs (n = 3). Scale bar: 20 μm. Phosphorylation of AMPK in BMDMs **G** or PBMC-derived macrophages **I** treated with AICAR (300 μM) for 15 min (n = 3). rHMGB1 (his tag) content in BMDMs **H** or PBMC-derived macrophages **J** (n = 3). (K) mRNA level of TNF-α, IL-6 and IL-1β in BMDMs (n = 3). Cells were pretreated with AICAR (300 μM) or AMPK inhibitor Compound C (CC, 20 μM) for 15 min and then co-cultured with rHMGB1 (his tag) (10 nM) for 1 h (E, F, H, I and J). A significant difference was revealed following unpaired Student’s t-test (C, G and I) or one-way ANOVA (A, B, F, H, J and K) (*p < 0.05, **p < 0.01 and ***p < 0.001 vs. Control; ^#^*p* < 0.05, ^##^*p* < 0.01 and ^###^*p* < 0.001 vs. AICAR group; Bonferroni post hoc tests)

To find a solution to clear HMGB1, macrophage phagocytosis has attracted our attention for the endocytosis of HMGB1. Recombinant HMGB1 (rHMGB1) (his tag) was used to evaluate macrophage phagocytosis. As shown in Fig. 2A, pretreatment with L-OHP (0.5, 1, or 2 h) decreased the amount of rHMGB1 (his tag) in mouse BMDMs after being co-cultured with rHMGB1 (his tag) for 60 min. Considering the regulatory effect of AMPK on macrophage phagocytosis [21, 22], we found that L-OHP decreased AMPK phosphorylation in mouse BMDMs (Fig. 2B) and in the DRG of CIPN mice (Fig. 2C). Compared with the Control group, the AMPK activator AICAR significantly increased the amount of rHMGB1 (his tag) in mouse BMDMs at 30 and 60 min after being co-cultured with recombination rHMGB1 (his tag) (Fig. 2D, E and F). Data in Fig. 2G and H show that AICAR increased the phosphorylation of AMPK in mouse BMDMs, and the increased amount of rHMGB1 (his tag) in the AICAR group was abolished by the AMPK inhibitor Compound C (CC). We further investigated whether AMPK-facilitated engulfment of HMGB1 was involved in regulating the inflammatory state of macrophages. As shown in Fig. 2K, AICAR decreased the mRNA levels of inflammatory factors (TNF-α, IL-1β, and IL-6) induced by rHMGB1 (his tag).

Furthermore, we collected human peripheral blood mononuclear cells (PBMC) from human blood and confirmed our conclusion in mouse BMDMs. Interestingly, as shown in Fig. 2I and J, we also found that AICAR increased the engulfment of HMGB1 by human PBMC-derived primary macrophages, which was abolished by the AMPK inhibitor CC.

These data suggest that the AMPK activator AICAR promotes the engulfment of HMGB1 and reduces inflammation in mouse BMDMs and human PBMC-derived primary macrophages.

### AMPK activation promotes HMGB1 engulfment and degradation in lysosomes in mouse BMDMs

**Fig. 3:**
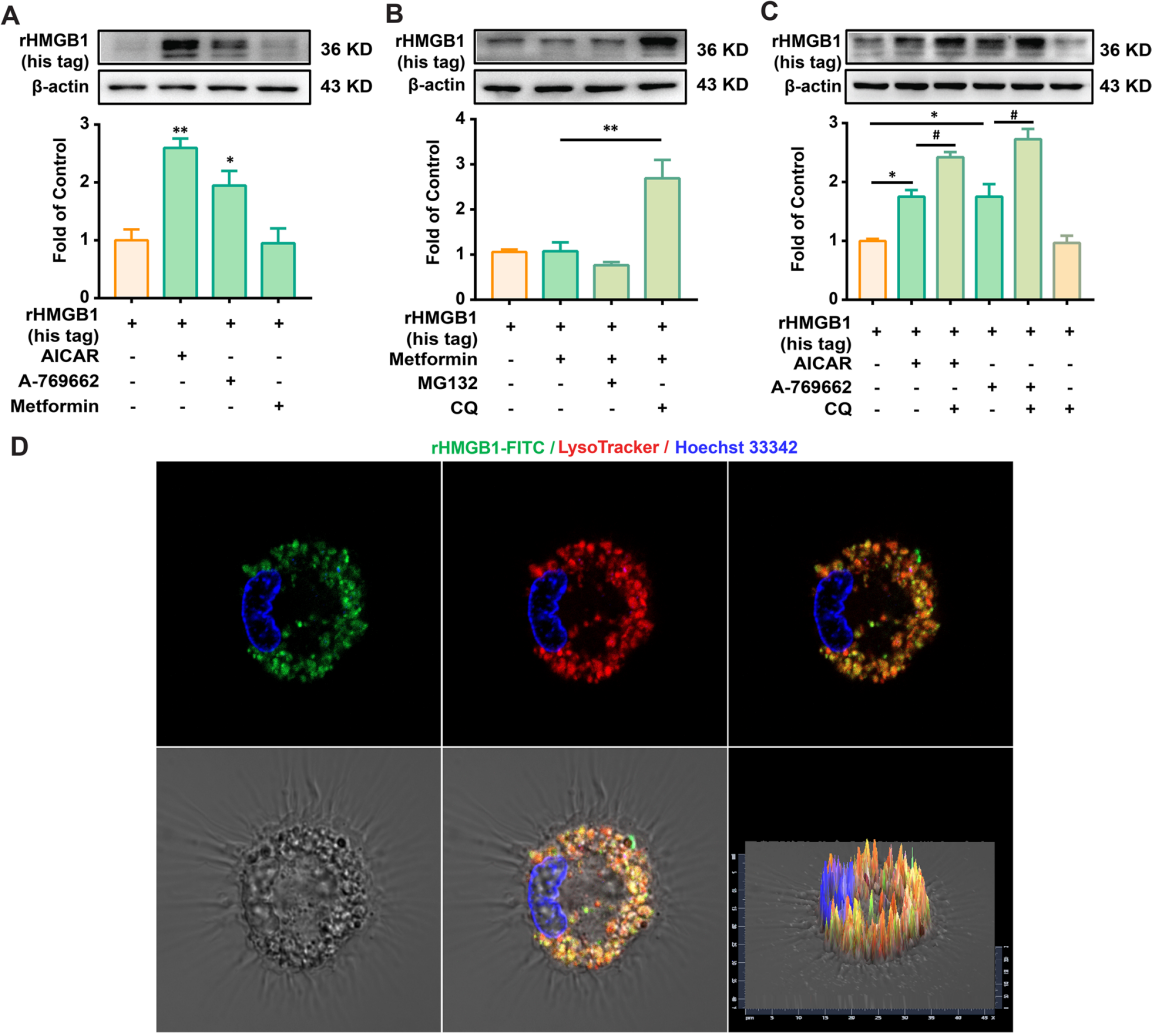
AMPK activation promotes engulfment of HMGB1 and facilitates degradation in lysosome in mouse BMDMs. **A** rHMGB1 (his tag) contents in BMDMs treated with AMPK activators AICAR (300 μM), A-769662 (100 μM), and metformin (2.5 mM) (n = 3). **B** rHMGB1 (his tag) contents in BMDMs treated with metformin (2.5 mM) in the presence of protease inhibitor (MG132, 10 μM) or lysosomal inhibitor (CQ, 20 μM) (n = 3). **C** rHMGB1 (his tag) contents in BMDMs treated with AICAR (300 μM) or A-769662 (100 μM) in the presence of CQ (20 μM) (n = 3). BMDMs were administered with inhibitor MG132 or CQ for 15 min, then treated with AMPK agonist (AICAR or metformin) for 15 min, followed by co-culturing with rHMGB1 (his tag) (10 nM) for 1 h, and subsequently collected for western blotting. **D** Confocal microscopy images of HMGB1-FITC (green) and Lysotracker (red) co-localization in BMDMs. Cells were pretreated with metformin (2.5 mM) for 15 min and then co-cultured with HMGB1-FITC (10 nM) and Lysotracker (75 nM) for 1 h. Scale bar: 20 μm. Representative bands and a data summary (n = 3 for each group) are shown. A significant difference was revealed following one-way ANOVA (*p < 0.05, **p < 0.01 and ***p < 0.001 vs. Control; ^#^*p* < 0.05, ^##^*p* < 0.01 and ^###^*p* < 0.001 vs. AICAR group or metformin group; Bonferroni post hoc tests)

Different AMPK activators (AICAR, A-769662, and metformin) were used to verify the effects of AMPK activation. As shown in Fig. 3A, AICAR and A-769662, but not metformin, significantly increased the amount of rHMGB1 (his tag). We speculated that rHMGB1 (his tag) endocytosed into mouse BMDMs in the metformin group may be degraded. Generally, protein degradation is mainly dependent on the lysosomal pathway; therefore, a protease inhibitor (MG132) and a lysosomal inhibitor (Chloroquine, CQ) were used. As shown in Fig. 3B and C, the lysosomal inhibitor, but not the protease inhibitor, markedly increased the amount of rHMGB1 (his tag) in the Metformin, AICAR, and A-769662 groups. We further investigated the co-localization of HMGB1 and lysosomes. Cells were pretreated with CQ to inhibit the degradation of lysosomes by HMGB1-FITC. As shown in Fig. 3D, HMGB1-FITC co-localized with lysosome 1 h after metformin administration. These data suggest that AMPK activation promotes HMGB1 engulfment and degradation in the lysosomes.

### AMPK activation increases the engulfment of HMGB1 by mouse BMDMs via the p38/SR-A1 signaling pathway

**Fig. 4:**
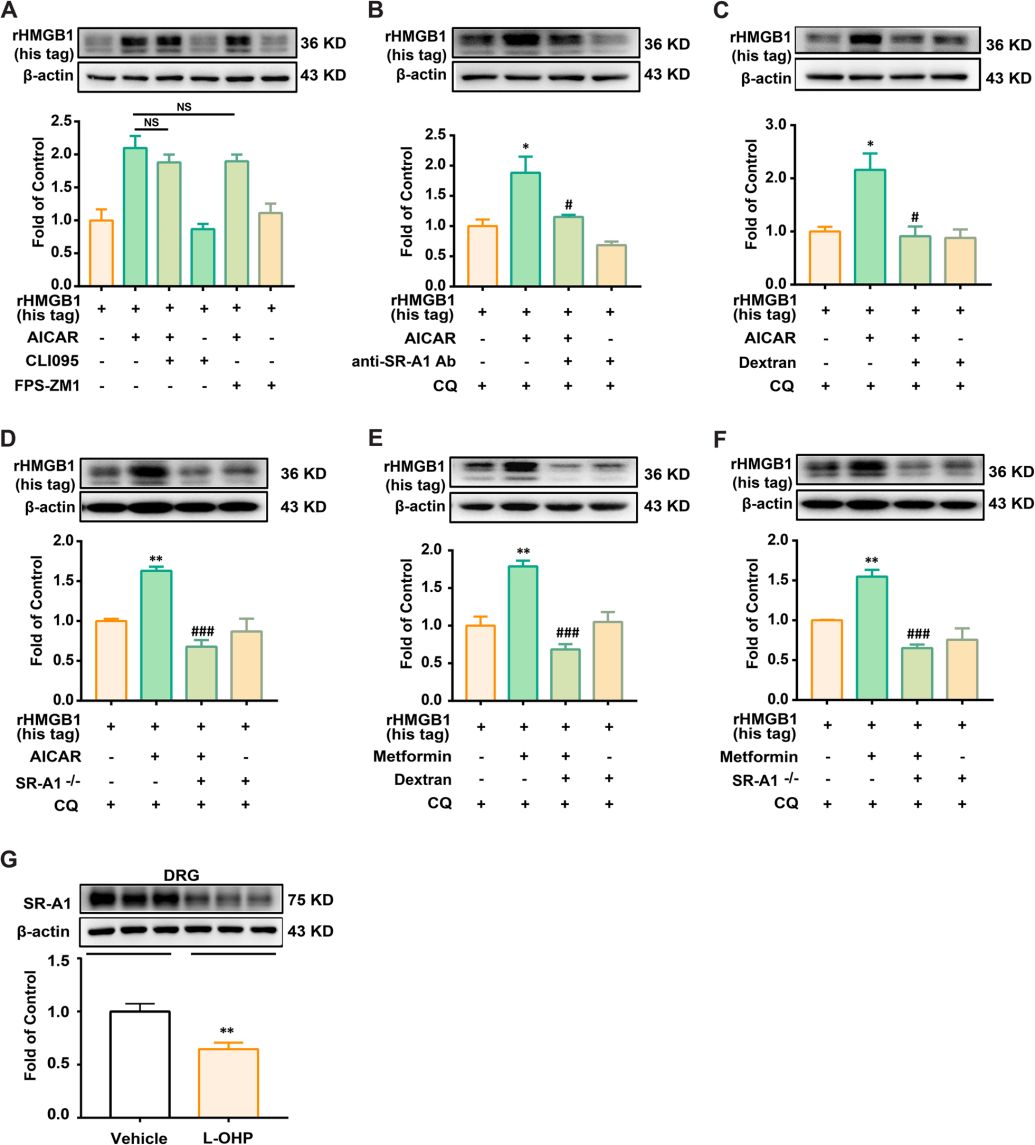
AMPK activation increases the engulfment of HMGB1 in an SR-A1-dependent manner in mouse BMDMs. **A** rHMGB1 (his tag) contents in BMDMs treated with AICAR (300 μM) in the presence of receptor-dependent endocytic pathway inhibitors (TLR4 inhibitor: CLI095, 10 μM; RAGE inhibitor: FPS-ZM1, 100 nM). rHMGB1 (his tag) contents in BMDMs treated with AICAR (300 μM) (**B**, **C** and **D**) or metformin (2.5 mM) (**E** and **F**) in the presence of anti-SR-A1 antibody (2 μg/ml), SR-A1 inhibitor dextran sulfate sodium salt (100 μg/ml) or SR-A1 KO (n = 3). **G** Phosphorylation of AMPK in the DRG of mice. DRG samples were collected on the 14th day after the first administration of L-OHP (3 mg/kg, *i.p.*) or vehicle (n = 3). BMDMs^SR−A1−/−^ (**D**) (**F**) were isolated from the bone marrow of SR-A1 KO mice. BMDMs were administered with/without CQ (20 μM) for 15 min, administered with inhibitors or anti-SR-A1 Ab for 15 min, then treated with AMPK agonist (AICAR or metformin) for 15 min, followed by co-culturing with rHMGB1 (his tag) (10 nM) for 1 h, and subsequently collected for western blotting. Representative bands and a data summary (n = 3 for each group) are shown. A significant difference was revealed following one-way ANOVA (*p < 0.05, **p < 0.01 and ***p < 0.001 vs. Control; ^#^*p* < 0.05, ^##^*p* < 0.01 and ^###^*p* < 0.001 vs. AICAR group or metformin group; Bonferroni post hoc tests)

Several classical endocytic pathways, such as receptor-mediated endocytosis, exist in macrophages [23]. To investigate the mechanism by which macrophages endocytose HMGB1, various endocytosis inhibitors were used. As shown in Fig. 4A, Cli095 (TLR-4 inhibitor) and FPS-ZM1 (RAGE inhibitor) did not inhibit the engulfment of HMGB1 promoted by AICAR.

We will continue to explore potential receptors. CQ was used to inhibit HMGB1 degradation. SR-A1 neutralizing antibody was used to inhibit SR-A1 function. Interestingly, we found that anti-SR-A1 Ab significantly decreased AICAR-induced engulfment of HMGB1 (Fig. 4B). We further used SR-A1 inhibitor dextran and SR-A1 KO mice-derived BMDMs to verify the effect of SR-A1. SR-A1 inhibition abolished the engulfment of HMGB1 induced by AICAR (Fig. 4C and D).

To confirm that AMPK activation increased the engulfment of HMGB1 in mouse BMDMs in an SR-A1-dependent manner, another AMPK activator, metformin, was employed. As shown in Fig. 4D, the SR-A1 inhibitor dextran and SR-A1 knockout abolished the engulfment of HMGB1 promoted by metformin in mouse BMDMs (Fig. 4E and F).

To verify the importance of MSR and its status in CIPN, we further evaluated the expression of SR-A1 in CIPN mice in vivo. Studies showed that numerous macrophages infiltrated the DRG of mice after L-OHP administration [24, 25]. SR-A1 is mainly localized in macrophages and central microglia [26]. Thus, we measured the expression of SR-A1 in the DRG of CIPN mice. As shown in Fig. 4G, L-OHP markedly decreased the expression of SR-A1 compared to that in the Vehicle Group.

**Fig. 5:**
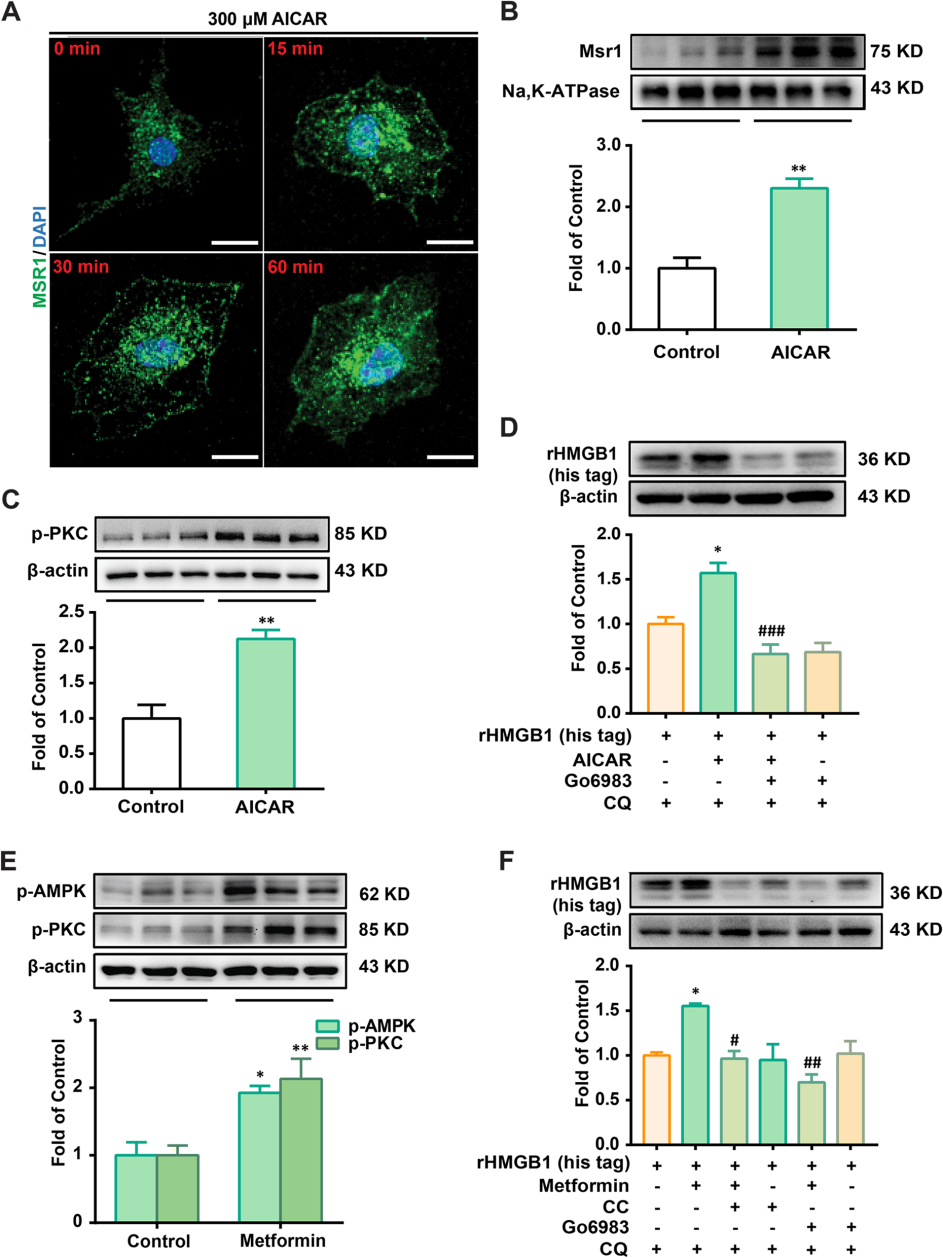
Activation of AMPK/PKC axis promoted SR-A1 transfer to cytomembrane for engulfment of HMGB1. **A** Time course of SR-A1 (green) location in BMDMs treated with AICAR (300 μM). Confocal microscopy images were captured at 0, 15, 30, and 60 min after AICAR administration. Scale bar: 10 μm. **B** The expression of SR-A1 on the membrane of BMDMs treated with AICAR (300 μM, 15 min) (n = 3). **C** Phosphorylation of PKC in BMDMs treated with AICAR (300 μM, 15 min) (n = 3). **D** rHMGB1 (his tag) contents in BMDMs treated with AICAR (300 μM) in the presence of PKC inhibitor (Go6983, 100 nM) (n = 3). **E** Phosphorylation of AMPK and PKC in BMDMs treated with metformin (2.5 mM, 15 min). **F** rHMGB1 (his tag) contents in BMDMs treated with AICAR (300 μM) in the presence of the AMPK inhibitor (CC, 20 μM) or PKC inhibitor (Go6983, 100 nM) (n = 3). BMDMs were administered with CQ (20 μM) for 15 min, then administered with inhibitors Go6983 (D) (F) or CC (F), then treated with AMPK agonist (AICAR or metformin) for 15 min, followed by co-culturing with rHMGB1 (his tag) (10 nM) for 1 h, and subsequently collected for western blotting. Representative bands and a data summary (n = 3 for each group) are shown. A significant difference was revealed following unpaired Student’s t-test (B and C) or one-way ANOVA (D, E and F) (*p < 0.05, **p < 0.01 and ***p < 0.001 vs. Control; ^#^*p* < 0.05, ^##^*p* < 0.01 and ^###^*p* < 0.001 vs. AICAR group or metformin group; Bonferroni post hoc tests)

We further explored the role of AMPK in SR-A1 activity. As shown in Fig. 5A and B, the co-localization of SR-A1 and cytomembrane increased after AICAR administration. In addition, AMPK activation (with AICAR and metformin) increased PKC phosphorylation (Fig. 5C and E). The PKC inhibitor Go6983 abolished the upregulation effect of AMPK activators (AICAR and metformin) on the engulfment of HMGB1 in mouse BMDMs (Fig. 5D and F).

These results indicate that the AMPK/PKC axis promotes SR-A1 translocation to the cytomembrane and mediates HMGB1 engulfment.

### Metformin activates AMPK and decreases HMGB1 accumulation to attenuate L-OHP-induced mechanical allodynia in mice model

**Fig. 6:**
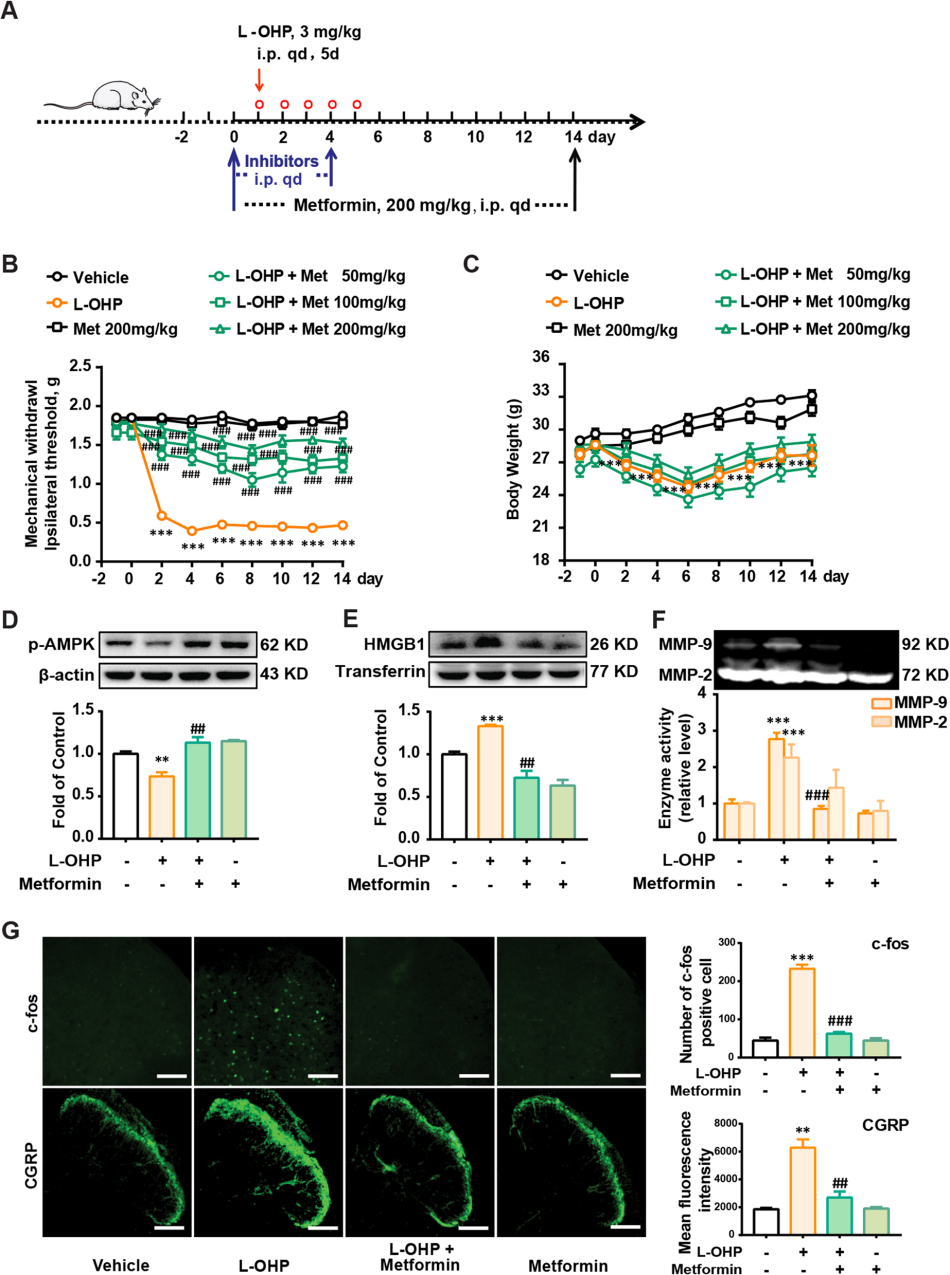
Metformin activates AMPK and decreases HMGB1 accumulation to attenuate L-OHP-induced mechanical allodynia. **A** The general scheme of experiments in vitro. Mechanical thresholds (**B**) and body weight (**C**) of CIPN mice treated with metformin (50, 100 and 200 mg/kg, *i.p.*) (n = 8). Phosphorylation of AMPK in DRG (**D**), HMGB1 content (**E**), and MMP-9/2 activity (**F**) in plasma of mice treated with metformin (200 mg/kg, *i.p.*) (n = 3). **G** Immunofluorescence analysis of neuronal c-fos and CGRP expression in the spinal cord of mice treated with metformin (200 mg/kg, *i.p.*) (n = 3). Mice were administered with L-OHP injection (3 mg/kg, *i.p.*, five consecutive days). Metformin (200 mg/kg, *i.p., q.d*) or vehicle was injected for 14 days, starting a day before the first injection of L-OHP. DRG (D), Plasma (**E** and **F**) and spinal cord (**G**) were collected after the final treatment of metformin for western blotting or immunofluorescence assay. The quantification of c-fos and CGRP immunofluorescence was respectively represented as the number of c-fos-positive cells and the mean fluorescence intensity of CGRP in the dorsal horn. Representative bands and a data summary (n = 4 for each group) are shown. Significant differences were revealed following one-way (D-G) or two-way (B and C) ANOVA (*p < 0.05, **p < 0.01 and ***p < 0.001 vs. Control; ^#^*p* < 0.05, ^##^*p* < 0.01 and ^###^*p* < 0.001 vs. L-OHP group; Bonferroni post hoc tests)

To determine whether AMPK activation could prevent L-OHP-induced mechanical allodynia, metformin was administered for 14 consecutive days at doses of 50, 100, or 200 mg/kg, of which the highest dose (200 mg/kg daily) was used to treat diabetic mice [27] and is efficacious in surgical nerve injury-induced neuropathic pain [28]. As shown in Fig. 6A, B and C, metformin prevented L-OHP-induced mechanical allodynia in a dose-dependent manner and did not affect the body weight of CIPN mice. Additionally, metformin increased AMPK phosphorylation in the DRG of CIPN mice (Fig. 6D) and decreased HMGB1 accumulation and MMP-9 activity in the blood of CIPN mice (Fig. 6E and F). We further measured the expression of CGRP (an indicator of pain) and c-fos (a marker of the activation of nociceptive neurons), and data showed that metformin decreased the expression of CGRP and c-fos in CIPN mice spinal cord (Fig. 6G). These data suggest that AMPK activation could decrease HMGB1 accumulation and MMP-9 activity in the blood and alleviate L-OHP-induced mechanical allodynia in a mouse model.

### SR-A1 is a key mediator of AMPK-mediated inhibition of HMGB1 accumulation and MMP-9 activity

**Fig. 7:**
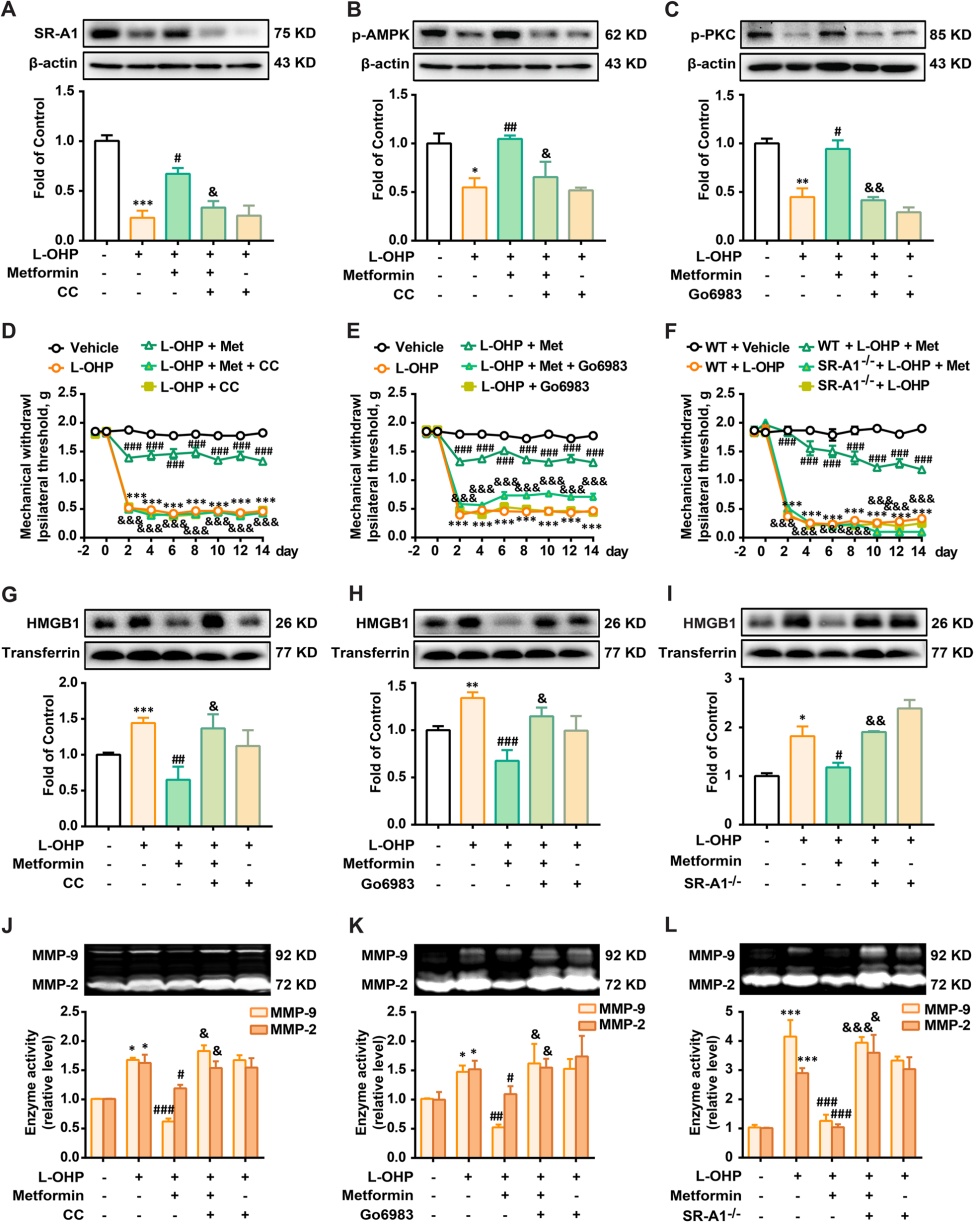
SR-A1 is essential for AMPK-mediated reduction of HMGB1 accumulation and MMP-9 activity. **A**–**C** The expression of SR-A1, p-AMPK or p-PKC in DRG of mice treated with metformin (200 mg/kg, *i.p.*) at present of AMPK inhibitor (CC, 25 mg/kg, *i.p.*) or PKC inhibitor (Go6983, 4 mg/kg, *i.p.*) (n = 3). Mechanical thresholds of mice treated with metformin (200 mg/kg, *i.p.*) in the presence of CC (**D**), Go6983 (**E**) or SR-A1 KO (F) (n = 8). HMGB1 content and MMP-9 activity in plasma of mice treated with metformin (200 mg/kg, *i.p.*) in the presence of CC (**G**, **J**) (n = 5), Go6983 (**H**, **K**) (n = 5) or SR-A1 KO (**I**, **L**) (n = 3). Mice or SR-A1 KO mice were administrated with L-OHP injection (3 mg/kg, *i.p.*, five consecutive days). Metformin (200 mg/kg, i.p. q.d) or vehicle was injected for 14 days, starting from a day before the first injection of L-OHP. CC and Go6983 were administered once daily for 5 days, 30 min before the administration of metformin. Plasma (**G**–**I**) and DRG (**A**–**C**) were collected after the final treatment of metformin for western blotting (**A**–**C**, **G**–**I**) or gelatin zymography (**J–L**). Representative bands and a data summary (n = 4 for each group) are shown. Significant differences were revealed following one-way or two-way ANOVA (*p < 0.05, **p < 0.01 and ***p < 0.001 vs. Control; ^#^*p* < 0.05, ^##^*p* < 0.01 and ^###^*p* < 0.001 vs. L-OHP group; ^&^*p* < 0.05, ^&&^*p* < 0.01 and ^&&&^*p* < 0.001 vs. L-OHP + metformin group; Bonferroni post hoc tests)

We further investigated the mechanism underlying AMPK activation by decreasing HMGB1 accumulation in vivo. The general scheme of experiments is shown in Fig. 6A. The AMPK inhibitor CC and PKC inhibitor Go6983 were administered to SR-A1 KO mice. As shown in Fig. 7A, AMPK activation increased the expression of SR-A1 in the DRG of CIPN mice, which was abolished by CC treatment. CC and Go6983 decreased the phosphorylation of AMPK and PKC in the DRG of CIPN + Met mice, respectively (Fig. 7B and C). CC, Go6983, and SR-A1 knockout abolished the analgesic effect of metformin (Fig. 7D–F) and abolished the reduction effects of metformin on HMGB1 accumulation and MMP-9 activity in the blood of CIPN mice (Fig. 7G–L). These data suggest that AMPK activation inhibits HMGB1 accumulation and MMP-9 activity via AMPK/SR-A1 signaling pathway in a mouse model in vivo.

## Discussion

The major findings of this study are as follows: (1) L-OHP significantly increased HMGB1 accumulation and MMP-9 activity in the plasma of CIPN patients and mice. (2) Plasma HMGB1 accumulation is positively correlated with VAS and L-OHP cumulative dose in patients with CIPN. (3) In mouse BMDMs, AMPK activation promoted engulfment of HMGB1 and degradation in lysosomes and decreased inflammatory factor expression via the p38/SR-A1 dependent signaling pathway. (4) In human PBMC-derived primary macrophages, AMPK activation increases HMGB1 engulfment. (5) In vivo, metformin decreased plasma HMGB1 accumulation and attenuated CIPN via the AMPK/p38/SR-A1 signaling pathway.

Our previous research demonstrated that L-OHP induced the release of HMGB1 from RAW 264.7 and SH-SY5Y cells, increased MMP-9/2 activity in the DRG, and participated in developing CIPN. Inhibition of MMP-9 activity mediated by NAC (N-acetylcysteine, an expectorant) has a therapeutic effect [12, 29]. Another study validates our conclusion in clinic and showes that NAC could reduce the incidence of the neuropathy induced by oxaliplatin and delay its occurrence in patients with gastric or colorectal cancers, although the mechanism has not been clarified [30]. In this study, for the first time, we found that in the plasma of patients and mice, L-OHP-induced mechanical allodynia was associated with increased HMGB1 accumulation and MMP-9/2 activity (Fig. 1A and B). Additionally, we found a positive correlation between plasma HMGB1 accumulation and VAS and plasma HMGB1 accumulation and L-OHP cumulative dose in patients with CIPN (Fig. 1C and D). Considering the important role of HMGB1, we further investigated the pathogenesis of CIPN from another perspective: scavenging HMGB1.

Is there an endogenous mechanism underlying HMGB1 clearance? Macrophages are known to act as ‘scavengers,’ clearing pathogens, bacteria, apoptotic cell debris, etc. [31]. Macrophages also play critical roles in HMGB1-mediated inflammatory response [32]. Evidence has confirmed that HMGB1 triggers dynamin-dependent endocytosis in macrophages [33]. The results showed that macrophages could endocytose HMGB1, which was significantly reduced by L-OHP treatment (Fig. 2A). Interestingly, the inhibition of HMGB1 engulfment was associated with a reduction in p-AMPK in the L-OHP group (Fig. 2B and C). However, the role of AMPK in the macrophage endocytosis of HMGB1 remains unclear. Since our previous study demonstrated that AMPK activation attenuates neuropathic pain in rats [19], we speculated that AMPK activation may attenuate L-OHP-induced peripheral neuropathy.

We investigated the relationship between AMPK and macrophage phagocytosis by HMGB1 cells. The data showed that AMPK activation significantly increased the engulfment of HMGB1 not only in mouse BMDMs but also in human PBMC-derived primary macrophages (Fig. 2D–I). We also found that AMPK activation decreased the mRNA levels of inflammatory cytokines induced by HMGB1 (Fig. 2J), suggesting that AMPK activation may decrease HMGB1 accumulation and inflammation. Additionally, two other AMPK activators were used for the experiments with mouse BMDMs (Fig. 3A). However, the AMPK activator, metformin, did not increase the amount of rHMGB1 (his tag) in BMDMs. Two possible mechanisms exist: (1) metformin does not promote rHMGB1 (his tag) internalization, and (2) metformin promotes rHMGB1 (his tag) internalization and degradation. The results showed that lysosomal inhibitors, but not protease inhibitors, could significantly inhibit the degradation of rHMGB1 (his tag) in BMDMs treated with metformin, AICAR, and A769662 (Fig. 3B and C). Combined with the lysotracker data (Fig. 3D), the results indicate that AMPK activation could increase the engulfment and degradation of HMGB1 in BMDMs lysosomes.

Therefore, it is necessary to explore the mechanism of HMGB1 engulfment in relation to AMPK. Macrophage endocytosis includes (TLRs, RAGE, SR, etc.)-mediated endocytosis [23, 34, 35]. Studies have shown that DAMPs, especially peroxiredoxin clearance in the brain by infiltrating mononuclear phagocytes, are beneficial for the resolution of sterile inflammation and treatment of stroke, where SR-A1 and HMGB1 are mentioned [16]. However, the study did not provide direct evidence that SR-A1 mediates the internalization of HMGB1 in vitro but showed that HMGB1 content in the brain was regulated by SR-A1 in vivo, suggesting the possibility that SR-A1 is involved in macrophage phagocytosis of HMGB1. In this study, we activated AMPK using agonists and performed further studies. We found that SR-A1 inhibitor, neutralizing antibody, and SR-A1 knockout significantly inhibited the engulfment of HMGB1 induced by AICAR or metformin (Fig. 4). Additionally, AMPK activation could promote SR-A1 translocation to the cytomembrane, the process by which PKC participates (Fig. 5).

It has been reported that HMGB1 endocytosis by macrophages could lead to lysosomal destabilization and macrophage pyroptosis via the RAGE-mediated signaling pathway [33]. In this study, we showed that the engulfment of HMGB1 promoted by AMPK activation was abolished by the lysosomal inhibitor CQ but not by the RAGE inhibitor FPS-ZM1 (Figs. 3C and 4B). Lysosomes maintained their morphology and co-localized with HMGB1 1 h after rHMGB1 (his tag) administration (Fig. 3D), suggesting that the AMPK/SR-A1 axis-mediated engulfment of HMGB1 may not induce macrophage pyroptosis.

We further verified the analgesic effects of AMPK activation on CIPN in vivo. The AMPK activator, metformin, attenuated L-OHP-induced mechanical allodynia, decreased HMGB1 accumulation and MMP-9 activity in CIPN mouse blood, and reduced the expression of CGRP (an indicator of pain) and c-fos (a marker of nociceptive neuron activation) in the spinal cord of CIPN mice (Fig. 6). In addition, considering that metformin is a substrate of OCT2 transporter as well as oxaliplatin, we measured the drug-drug interaction (competition for OCT2 transport) happened between metformin and oxaliplatin. The data was shown in Additional file 1: Fig. S1, metformin did not affect the level of L-OHP in the DRG of mice. We also investigated the mechanism underlying the analgesic effect of metformin. AMPK activation increased the expression of SR-A1 in the DRG of mice. AMPK inhibition, PKC inhibition, and SR-A1 knockout abolished the suppressive effects of metformin on mechanical allodynia, HMGB1 accumulation, and MMP-9 activity (Fig. 7) in CIPN mice in vivo.

## Conclusion

**Fig. 8:**
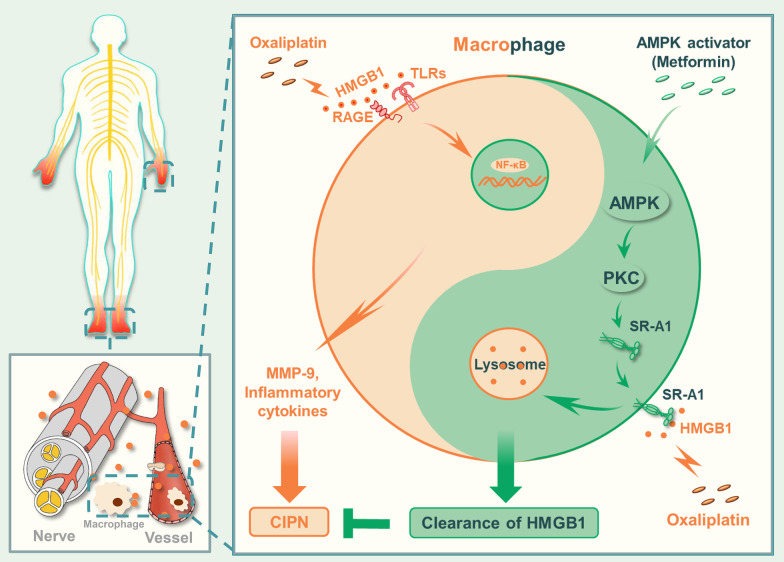
Schematic model indicates that promoting SR-A1-mediated engulfment of HMGB1 by AMPK activation facilitates the resolution of inflammation and alleviates CIPN. L-OHP increases HMGB1 release from stress cells and inhibits macrophage endocytosis of HMGB1 via AMPK inactivation. HMGB1 accumulation subsequently induces MMP-9 release via the TLR4-mediated inflammatory signaling pathway and contributes to the progression of CIPN. AMPK agonists activate PKC and promote SR-A1 transfer to the membrane to mediate the engulfment of HMGB1 and degradation in lysosome, leading to the resolution of inflammation and attenuation of CIPN

In summary, we demonstrated that HMGB1 plays an important role in CIPN in animal experiments and clinics and provides metformin as a potential treatment for CIPN, which could activate the AMPK/p38/SR-A1 axis to promote HMGB1 clearance (Fig. 8).

## Supplementary Information

**Additional file 1**: Patients baseline characteristics and the level of platinum in DRG of mice.

**Additional file 2**: Raw data.

## Data Availability

All data associated with this study are presented in the paper and/or Additional file 2 showed as Raw data.

## Abbreviations

CIPN: Chemotherapy-induced peripheral neuropathy
HMGB1: High mobility group box 1
VAS: Visual analog scale
AMPK: AMP-activated protein kinase
SR-A1: Macrophage scavenger receptor A1
L-OHP: Oxaliplatin
PKC: Protein kinase C
PBMC: Peripheral blood mononuclear cells
BMDMs: Bone marrow-derived macrophages
MMP: Matrix metalloproteinase
