# Supplementary material for: Promoting AMPK/SR-A1-mediated clearance of HMGB1 attenuates chemotherapy-induced peripheral neuropathy
Source: Cell Commun Signal. 2023 May 4;21:99. doi: 10.1186/s12964-023-01100-9 (PMC10161452; doi:10.1186/s12964-023-01100-9)
Supplement: Supplementary file 2 — Additional file 1: Patients baseline characteristics and the level of platinum in DRG of mice. [file 12964_2023_1100_MOESM2_ESM.docx]

**Promoting AMPK/SR-A1-mediated clearance of HMGB1 attenuates chemotherapy-induced peripheral neuropathy**

Xing Yang ^1*^, Rumeng Jia ^1*^, Fan Hu ^1*^, Wen Fan ^1*^, Tongtong Lin ^1^, Xiaotao Zhang ^2^, Chenjie Xu ^3^, Shirong Ruan ^1^, Chunyi Jiang ^1^, Yan Li ^4^, Cailong Pan ^1^, Yang Yang^5🖂^,Liang Hu ^1🖂^, Qi Chen ^6🖂^, Wen-Tao Liu ^1🖂^

* These authors contributed equally to this work.

^🖂^ Addressed correspondence to

lianghu@njmu.edu.cn, Department of Pharmacology, School of Basic Medical Sciences, Nanjing Medical University, Nanjing 211166, China

Or

Jszlyyyy @163.com, Department of Anesthesiology, The Affiliated Cancer Hospital of Nanjing Medical University & Jiangsu Cancer Hospital & Jiangsu Institute of Cancer Research, Nanjing, Jiangsu 211166, China

Or

qichen@njmu.edu.cn, Atherosclerosis Research Center, Key Laboratory of Cardiovascular Disease and Molecular Intervention, Nanjing Medical University, Nanjing, Jiangsu 210029, China

Or

painresearch@njmu.edu.cn, Department of Pharmacology, School of Basic Medical Sciences, Nanjing Medical University, Nanjing 211166, China

Co-authors' email addresses

Xing Yang: xingyang@njmu.edu.cn; Rumeng Jia: 1034006921@qq.com; Fan Hu: [hufan@njmu.edu.cn; Wen](mailto:hufan@njmu.edu.cn;%20Wen) Fan:fanwennjmu@163.com; Tongtong Lin: 925703439@qq.com; Xiaotao Zhang: 18669710019@126.com; Chenjie Xu: xcj2001@126.com; Shirong Ruan: 1783793331@qq.com; Chunyi Jiang: jcy@njmu.edu.cn; Yan Li: liyan16766@163.com; Cailong Pan: pancailong@njmu.edu.cn; Yang Yang: Jszlyyyy@163.com; Liang Hu: lianghu@njmu.edu.cn; Qi Chen: qichen@njmu.edu.cn; Wen-tao Liu: painresearch@njmu.edu.cn.

| **Table S1. Patients baseline characteristics** | | |
| --- | --- | --- |
| Characteristic, n (%) |  | Group  (n=20) |
| Age(years) | <=50 | 2 |
|  | 51-60 | 3 |
|  | 61-70 | 12 |
|  | >=71 | 3 |
|  |  |  |
| Gender | Male | 13 |
|  | Female | 7 |
|  |  |  |
| Clininal diagnoses, n | Colon cancer | 11 |
|  | Rectal cancer | 9 |
|  |  |  |
| Chemotherapy agents | XELOX | 10 |
|  | FOLFOX4 | 2 |
|  | FOLFOX6 | 5 |
|  | SOX | 1 |
|  | Oxaliplatin | 1 |
|  | XELOX，FOLFOX6 | 1 |
| Total of oxaliplatin | <500  500-1000  1001-2000 | 7  6  7 |
|  |  |  |
| Vas score | 3-5 | 10 |
|  | 6-7 | 6 |
|  | 8-9 | 4 |

**Table S2. Sequences of primers for real-time quantitative polymerase chain reaction**

| Gene |  | Primer sequences |
| --- | --- | --- |
| IL-1β | Forward | 5′-TCATTGTGGCTGTGGAGAAG-3’ |
|  | Reverse | 5′-AGGCCACAGGTATTTTGTCG-3’ |
| TNF-α | Forward | 5′-CATCTTCTCAAAATTCGAGTGACAA-3’ |
|  | Reverse | 5′-TGGGAGTAGACAAGGTACAACCC-3’ |
| IL-6 | Forward | 5′-CTGCAAGAGACTTCCATCCAG-3’ |
|  | Reverse | 5′-AGTGGTATAGACAGGTCTGTTGG-3’ |
| ACTIN | Forward | 5′-GTGACGTTGACATCCGTAAAGA-3′ |
|  | Reverse | 5′-GCCGGACTCATCGTACTCC-3′ |

Figure S1


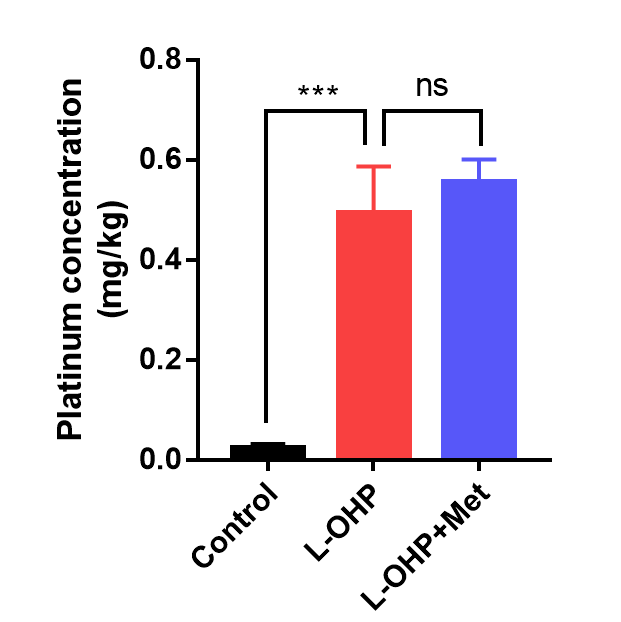


Fig. s1 The level of platinum in DRG of mice. DRG samples were collected on the 7th day after the first administration of L-OHP (3 mg/kg, *i.p.*) or vehicle. The Platinum concentration was detected by inductively coupled plasma emission mass spectrometer (ICP-MS 7800) (n=4). Significant differences were revealed following one-way ANOVA (*p < 0.05, **p < 0.01 and ***p < 0.001 vs. Control; Bonferroni post hoc tests).
